# The Link Between Masculinity and Spatial Skills Is Moderated by the Estrogenic and Progestational Activity of Oral Contraceptives

**DOI:** 10.3389/fnbeh.2021.777911

**Published:** 2022-01-27

**Authors:** Adriene M. Beltz, Amy M. Loviska, Dominic P. Kelly, Matthew G. Nielson

**Affiliations:** ^1^Department of Psychology, University of Michigan, Ann Arbor, MI, United States; ^2^Department of Human Development and Family Studies, Purdue University, West Lafayette, IN, United States

**Keywords:** androgenic activity, estrogenic activity, progestational activity, mental rotation (MR), masculinity, femininity, hormonal contraceptive, ovarian hormones

## Abstract

Conversations about gender and spatial skills frequently dissolve into a hackneyed debate over nature and nurture. This is particularly true for conversations concerning three-dimensional (3D) mental rotations skill, which shows the largest gender difference of all aspects of cognition, with men—on average—outperforming women. To advance this empirical area of inquiry, biopsychosocial influences on spatial skills should be considered, and a unique opportunity do to that is provided by combined oral contraceptives (OCs). OCs with relatively low estradiol doses and with highly androgenic progestins have been positively related to spatial skills. Gender self-concepts, including masculine and feminine self-perceptions, have also been positively related to spatial skills. It is wholly unknown, however, whether the exogenous sex hormones contained in OCs moderate the link between self-perceived masculinity and 3D mental rotations. This study filled that knowledge gap by utilizing a sample of 141 naturally cycling (NC) women and 229 OC users who completed a computerized survey and cognitive tests. A series of moderation analyses examined whether the link between masculinity and 3D mental rotations depended on pill use or on the estrogenic, progestational, or androgenic activity in OCs, which were operationalized using a novel coding scheme. Results showed that the positive masculinity-3D mental rotations link was only present for NC women, presumably because it was altered by the exogenous hormones in OCs. Indeed, the link was accentuated in users of OCs with relatively low estrogenic and high progestational activity. Future research on menstrual cycle and pill phase is needed, but these findings importantly delineate ways in which biological and psychosocial factors combine to explain variation in spatial skills among women. They also suggest that focus should be placed on the under-investigated progestational activity of OCs, which is facilitated by the novel quantification of OC action used in this study. Thus, this research increases understanding of the neurocognitive and behavioral correlates of ovarian hormones and has implications for the betterment of women’s health.

## Introduction

Interest in the biopsychosocial correlates of gender differences in spatial skills has been persistent across time and pervasive across scientists, parents, educators, and policy makers (see Newcombe, 2020). There is particular interest in three-dimensional (3D) mental rotations skill in which men outperform women on average, despite substantial variability within each gender (Voyer, 2011; Halpern, 2013; Beltz et al., 2020). Sex hormones, such as androgens and estradiol, have been consistently shown to be biological contributors to this gender difference. For instance, prenatal androgens facilitate spatial skills throughout life, and the gender difference in mental rotations skill is reduced during low estradiol phases of the menstrual cycle (Berenbaum et al., 2012; Peragine et al., 2020). There are also several psychosocial contributors to gender differences in spatial skills, including parent socialization (e.g., use of spatial language; Pruden and Levine, 2017) and a person’s own gender self-concept, or how they think about and use gender labels like *masculine* and *feminine* (Nash, 1979; Reilly and Neumann, 2013). Despite emerging evidence and hypotheses concerning the gendered and interactive nature of biological and psychosocial influences on cognition (e.g., Hausmann et al., 2009; Berenbaum et al., 2018), research that concurrently examines these influences with respect to spatial skills is limited. Combined oral contraceptive (OC) users provide a unique opportunity to fill this knowledge gap because their pills contain exogenous estradiol and progestins that vary in androgenicity, yet they do not have different gender self-concepts (e.g., self-perceived femininity and masculinity) from naturally cycling (NC) women (Nielson and Beltz, 2021). Thus, the goal of this study was to determine whether ovarian hormonal milieu (marked by NC vs. OC status and the hormone activity of OCs) moderates links between women’s self-perceived masculinity and mental rotations skill.

There is strong empirical support for a positive association between masculinity and spatial skills. Indeed, the *sex-role mediation hypothesis* attempts to explain some mean-level gender differences in cognition through this purported mechanism (Nash, 1979), such that masculinity is positively related to spatial skills, whereas femininity is positively related to verbal skills. Empirical evidence from meta-analyses and well-powered studies (*N* > 300) has borne out this general pattern of results for masculinity and spatial skills, but findings are less consistent for femininity and verbal skills (Signorella and Jamison, 1986; Reilly and Neumann, 2013; Reilly et al., 2016; Kelly and Beltz, under review). The most frequently assessed spatial skill in the extant literature is 3D mental rotations skill, which shows the largest effects, and masculinity is often assessed by stereotypical gendered personality traits (e.g., “assertive” or “independent”; Bem, 1974), but also by self-perceptions of masculinity (e.g., “How masculine is your personality?”; Storms, 1979).

Although there is empirical support for an association between masculinity and spatial skills, the extant literature is unclear concerning potential gender differences in this link. This is somewhat surprising because the link between masculinity and spatial skills is an explanatory mechanism for cognitive gender differences, according to the sex-role mediation hypothesis. For instance, some studies report stronger relations between masculinity and spatial skills for men than for women (Reilly and Neumann, 2013), and other studies report that the relation is stronger for women than for men (Signorella and Jamison, 1986; Kelly and Beltz, under review).

This mixed evidence for a gender difference is not entirely surprising, though, because masculinity and spatial skills are complex, multi-determined constructs (Ruble et al., 2006; Verdine et al., 2017). This is apparent in the conceptualization of the sex-role mediation hypothesis and in work examining it. For instance, Nash (1979) suggested that cultural norms surrounding gender impact masculinity, and this predisposes participation in gender stereotypical activities that hone spatial skillsets. Consistent with that notion, recent work shows that gendered experiences and interests (operationalized as the extent to which college majors have a science, technology, engineering, and mathematics, or STEM, focus) partially explain the relation between masculinity and spatial skills, but only for women and only for particular skills (e.g., 3D mental rotations; Kelly and Beltz, under review). Thus, there is a pressing need for research that explicates the relation between masculinity and spatial skills—not just by identifying which social factors underlie it, but also by identifying which biological factors might qualify it. Indeed, the multidimensionality of gender (Ruble et al., 2006) and a compelling literature on sex hormone contributions to spatial skills suggest biology could play a significant role (reviewed in Beltz et al., 2020).

A unique opportunity to study biological, particularly neuroendocrinological, associations with masculinity and spatial skills is afforded by women who use OCs, which are a widely used natural experiment for ovarian hormone manipulations (see Beltz and Moser, 2020; Hampson, 2020). All OCs contain a progestin (synthetic progesterone), and combined OCs also contain a synthetic estrogen (typically ethinyl estradiol; Beltz and Moser, 2020; Hampson, 2020). Progestins in OCs vary (e.g., there are at least 12 types), and they have hormonal activity other than progestational activity, with degrees of androgenic or anti-androgenic activity most frequently studied (Beltz and Moser, 2020; Hampson, 2020). Most OC regimens attempt to mimic the average menstrual cycle, with about 21 active pills (containing a progestin and perhaps ethinyl estradiol) followed by about seven placebo pills, instigating menses. Monophasic OCs have consistent progestin and ethinyl estradiol doses in all active pills, whereas biphasic and triphasic OCs typically have consistent ethinyl estradiol doses but progestin doses that increase once or twice, respectively, across active pill days. Through neuroendocrine feedback mechanisms, OCs effectively halt ovulation, both preventing pregnancies and easing menstrual cycle-induced symptoms (e.g., dysmenorrhea). Thus, OCs also reduce *endogenous* levels of estradiol and progesterone in the body, while increasing levels of the *exogenous* hormones contained in the pills (Hampson, 2020). Though enlightening, comparisons between endogenous and exogenous hormone activity in OC users vs. NC women are not straight-forward because assays for the exogenous hormones contained in OCs are not widely available.

Nonetheless, comparisons between OC users and NC women are effective at marking whether general ovarian hormonal milieus are related to behavior and neurocognition (consistent with the approach of others; e.g., Peragine et al., 2020). These comparisons also indicate whether there may be differences between women who do and do not use OCs, as self-selection into OC use has been posited as a potential confound of research using NC-OC comparisons to make inferences about ovarian hormone influences (Oinonen et al., 2008). The limited research comparing NC women and OC users (that included women from this sample) indicates that groups do not differ in Big Five personality traits (i.e., neuroticism, extraversion, openness, agreeableness, and conscientiousness; Beltz et al., 2019), nor in gendered personality qualities most closely tied to the sex-role mediation hypothesis, such as instrumentality and expressivity as well as self-perceptions of masculinity and femininity (Nielson and Beltz, 2021). Moreover, these studies showed that the androgenicity of progestins, which vary across different types of OCs, did not impact results (Beltz et al., 2019; Nielson and Beltz, 2021). Progestin androgenicity is the extent to which progestins have androgenic properties and actions that are primarily due to their structural derivatives. Thus, studies of ovarian hormone links to gender self-concept suggest OC users of various pill formations do not differ from NC women.

Similarly for spatial skills, many studies do not report differences between OC users and NC women when OC users are considered as a single, heterogeneous group (Rosenberg and Park, 2002; Islam et al., 2008; Mordecai et al., 2008; Wharton et al., 2008; Puts et al., 2010; Griksiene and Ruksenas, 2011; Beltz et al., 2015). One explanation for the lack of differences is that research including NC women is challenged by menstrual cycle phase. Although endogenous hormone levels fluctuate throughout the cycle (i.e., with low estradiol and progesterone during menses, heightened estradiol during the follicular phase, followed by ovulation and slightly decreasing estradiol levels and concomitant peak in progesterone during the luteal phase), extant research examining how those phases are related to spatial skills is inconsistent, likely owing to poor research methodology. Some studies report improved spatial skills, especially mental rotations performance, during the follicular phase (e.g., Hausmann et al., 2000; McCormick and Teillon, 2001; Maki et al., 2002), but others do not (e.g., Mordecai et al., 2008; Griksiene and Ruksenas, 2011). These studies have small samples (most *N* < 40) and largely use unreliable count methods (e.g., estimating days since menses) to determine menstrual cycle phase (see Hampson, 2020; Gloe et al., under review). Another explanation for the lack of differences when OC users are considered as a single, heterogeneous group is that the variable hormone activities of OCs “cancel out” (e.g., the effects of androgenic and anti-androgenic progestins; Pletzer and Kerschbaum, 2014).

There is, however, indication that OC users and NC women differ in spatial skills, particularly 3D mental rotations skill, when OC users are categorized into smaller, homogenous groups informed by their pharmacokinetics (described in Beltz et al., 2015). These groups have primarily been formed based upon progestin androgenicity, as androgens have been shown to facilitate spatial skills in other natural experiments (reviewed in Beltz et al., 2020). Increasingly precise progestin-linked groups have led to increasingly precise inferences about OC influences on spatial skills. For instance, groups based solely on progestin generation, or the timing of a specific progestin’s introduction to the United States or European markets (Petitti, 2003), have shown mixed results: Studies reported no differences between NC women and androgenic generation or antiandrogenic generation OC users (Griksiene and Ruksenas, 2011) as well as increased spatial skills for androgenic early generation OC users compared to antiandrogenic newer generation users (Gurvich et al., 2020). Including information about progestin dose along with generation led to consistent inferences, though, with studies reporting that NC women outperformed monophasic antiandrogenic new generation OC users in mental rotations (Wharton et al., 2008; Griksiene et al., 2018), potentially suggesting that endogenous androgens in NC women contribute to performance. Finally, groups based on even more complete information about OC pharmacokinetics, including the exact type of progestin and estrogen as well as phase [according to Food and Drug Administration (FDA) criteria; USDHHS, 2018], revealed even more specific neuroendocrine effects: Women using monophasic pills containing ethinyl estradiol and the moderately androgenic progestin norethindrone acetate outperformed NC women and women using triphasic pills containing ethinyl estradiol and the mildly androgenic progestin norgestimate on a 3D mental rotations test (Beltz et al., 2015). This study using FDA criteria was also unique among OC studies in exploring exogenous hormone doses in OCs, finding that ethinyl estradiol dose was inversely related to mental rotations performance, but that progestin dose was not related to cognition (Beltz et al., 2015).

Thus, findings across OC studies converge in suggesting that there is a significant link between progestin androgenicity and spatial skills, especially mental rotations skill; ethinyl estradiol dose may also be important. These findings, however, overwhelmingly come from studies focused solely on the androgenicity of progestins, leaving unanswered questions about the roles of hormones that largely prevent ovulation, that is, the degree of estrogenic and progestational activity in different OCs. This may be an unfortunate byproduct of the methods researchers have used to account for OC heterogeneity, which rely almost exclusively on homogenous groups based on progestin generation or type. This approach certainly has benefits, but even these groups are not consistently defined across studies. Thus, future research that addresses heterogeneity in OCs without dropping information about degree of hormone activity, especially estrogenic and progestational activity, is sorely needed.

Despite a biological literature linking ovarian hormones (e.g., progestin androgenicity) to spatial skills and a psychosocial literature linking masculinity to spatial skills, the two perspectives have yet to be combined. Thus, the goal of this study was to take a biopsychosocial approach to the study of spatial skills, particularly 3D mental rotations skill, in NC women and OC users. Specifically, the link between self-perceived masculinity and mental rotations skill was examined in all women, and then potential moderation of the link by hormonal milieu was investigated to determine if its direction or magnitude differed for NC women and OC users. Menstrual cycle phase was not considered due to its unreliable determination in cross-sectional data (see Hampson, 2020; Gloe et al., under review), but the pharmacokinetics of OCs (i.e., estrogenic, progestational, and androgenic activity, according to levels reported in Dickey, 2020) were examined as moderators of the masculinity-mental rotations link within OC users, leveraging a novel four-point coding scheme. These activities “are dependent on the biological activities and the doses of individual estrogen and progestin components and by potentiating and antagonistic effects of one steroid component upon the other” (Dickey, 2020, p. 25). Based on past research, it was expected that the link would differ for NC women and OC users due to the progestin androgenicity of pills.

## Materials and Methods

Previous reports use some data from this sample (e.g., to show that NC women and OC users do not differ in personality or gender self-concept; Beltz et al., 2019; Nielson and Beltz, 2021), but study variables have not been investigated in concert, and the coding scheme for examining unique effects of estrogenic, progestational, and androgenic activity in OCs is novel. All participants provided informed consent before contributing to this research, which was conducted under the auspices of the University of Michigan Institutional Review Board.

### Participants

Participants were 370 women aged 18–28 years (*M*_*age*_ = 20.54 years; *SD*_*age*_ = 2.28) recruited from a United States university community via an established subject pool, online announcements, and posted flyers. Most identified as White (74%) and non-Hispanic (93%), with some identifying as Asian (16%), Black/African American (8%), or multiracial (2%). They were selected for inclusion in the current analyses from the full sample of 473 women and 221 men. Men were excluded from analyses because the primary research question on the role of ovarian hormone milieu in the link between behavior and cognition only concerned women. Women were excluded from analyses for the following reasons: having a reproductive health or medical condition that could impact ovarian hormone milieu, including past pregnancy, irregular menstrual cycles, the use of hormone-containing medications other than OCs (*n* = 64), being inattentive during data collection, including sleeping (*n* = 14) or failing to follow directions on the mental rotations test (*n* = 19), or being statistical outliers (i.e., three standard deviations from the sample mean) on age (*n* = 6); outliers bias error terms and age is related to masculinity and especially to femininity (Barrett and White, 2002; Jones et al., 2011).

Women in the analytic sample were grouped according to whether they were naturally cycling (*n* = 141) or using OCs (*n* = 229). OC users had been using the same pill for at least 3 months, and NC women had not used any hormonal contraceptive for at least 3 months and had regular menstrual cycles. OC users and NC women did not differ significantly in age, *t*(368) = −1.36, *p* = 0.174, or ethnicity, χ^2^(1) = 0.25, *p* = 0.615, but there were disproportionately more Asian identified women in the NC (26%) vs. OC group (11%), χ^2^(4) = 21.02, *p* < 0.001.

The OCs used by women in the sample represented a wide range of formulations. All included ethinyl estradiol, but progestins ranged from the highly androgenic levonorgestrel (*n* = 15) and norethindrone acetate (*n* = 71) to the mildly androgenic norgestimate (*n* = 71) to the antiandrogenic drospirenone (*n* = 45), among a variety of others (*n* = 18). Importantly, however, OCs were not merely grouped according to progestin types, but rather, they were coded according to their estrogenic, progestational, and androgenic activity, which are based on hormone types and doses, particularly their biological (e.g., receptor) actions (Dickey, 2020). Each hormone was considered to have *low*, *low-intermediate*, *intermediate-high*, or *high* activity in each OC formulation based on assays conducted in rodent and human tissues; estrogenic activity was determined by mouse uterine assay, progestational activity was determined by human endometrial response, and androgenic activity was determined by rat prostate assay (detailed in Dickey, 2020). These levels were converted into a four-point Likert scale to facilitate their inclusion in quantitative analyses (1 = *Low*, 2 = *Low-intermediate*; 3 = *Intermediate-high*, and 4 = *High*). For example, the commonly used OC Loestrin FE 1/20 (ethinyl estradiol, norethindrone acetate) had activity codes of estrogenic = 2, progestational = 3, and androgenic = 4, the triphasic Ortho Tri-Cyclen (ethinyl estradiol, norgestimate) had activity codes of estrogenic = 4, progestational = 1, and androgenic = 2, and Yaz (ethinyl estradiol, drospirenone) had activity codes of estrogenic = 2, progestational = 4, and androgenic = 1.

Nine OC users had pill types that could not be coded because their specific formulations were not contained in the coding system (Dickey, 2020). For the remaining 220 OC users (which makes this study among the largest—if not the largest—in terms of OC users with defined pharmacokinetic pill properties; see Warren et al., 2014), average estrogenic activity was 2.67 (*SD* = 1.03), progestational activity was 2.66 (*SD* = 1.29), and androgenic activity was 2.50 (*SD* = 1.18), and all activity levels ranged from 1 to 4, suggesting notable variability. Moreover, the activity levels showed both overlap and distinction, with estrogenic and progestational activity correlated at *r*(218) = −0.64, *p* < 0.001, estrogenic and androgenic activity at *r*(218) = −0.36, *p* < 0.001, and progestational and androgenic activity at *r*(218) = 0.11, *p* = 0.114. Thus, estrogenic and progestational activity were most highly related in this sample, but their relation only resulted in 41% overlap between the codes.

### Procedures

Participants came to a university research laboratory for an hour-long test session. During the session, they provided informed consent, described their reproductive history in a brief interview, recorded information about their OC formulation from the pill packet they brought with them (if applicable), and completed a monitored online survey. They were compensated with either course credit or $15.

### Measures

Participants responded to the questionnaire and completed the two cognitive tests described below as part of this study’s survey. All responses were provided on laboratory computers.

#### Masculinity

Self-perceived masculinity was assessed with the six-item Sex Role Identity Scale (Storms, 1979). This measure assesses gender self-concept, specifically gender self-labels, as items concern the extent to which individuals feel masculine or feminine in general, in their behavior, and in their dress. Specifically, three items correspond with masculinity (e.g., “How masculine is your personality?”), and three items correspond with femininity (e.g., “How feminine do you act, appear, and come across to others?”). Participants were asked to respond to each item on a five-point scale (1 = *Not at all* to 5 = *Extremely*). Following recent studies and current conceptualizations of gender as a continuum (Beltz, 2018; Gülgöz et al., 2019; Beltz et al., 2021), the three feminine items were reverse coded and averaged with the three masculine items, so that high scores reflect masculinity and low scores reflect femininity. Cronbach’s α for the scale was excellent at 0.89.

#### 3D Mental Rotations Skill

3D mental rotations skill was assessed with the Vandenberg and Kuse test (Vandenberg and Kuse, 1978). Each of 20 items consists of a 3D object composed of small blocks portrayed in 2D space and four response options that were similarly composed. Two of the response options were the same shape as the target but rotated in 3D space, which participants were instructed to identify. Participants had 10 min to complete the test. Following the “single scoring” procedure, participants received a single point for each correct response; thus, scores could range from 0 to 40.

#### General Cognition

General cognition was assessed with the advanced vocabulary test (Ekstrom et al., 1976), which is significantly correlated with overall intelligence (Sattler and Ryan, 2009). General cognition is important to consider in studies of spatial skills to isolate those skills from other aspects of cognition; this has been done in some previous neuroendocrine research (e.g., Beltz et al., 2015) and in research on the sex-role mediation hypothesis (Kelly and Beltz, under review). Each of 36 items consists of a target word or phrase and five response options. Participants were instructed to select which option is the best synonym of the target word, and they had 4 min to complete each half of the test. They received one point for each correct response, and were deducted a quarter point for each incorrect response; thus, scores could range from −9 to 36.

### Analysis Plan

Analyses were conducted in three parts. First, a regression was used to examine the relation between self-perceived masculinity and 3D mental rotations skill for all women. Second, a moderation analysis was used to examine whether the masculinity-mental rotations relation differed between NC women (coded 0) and OC users (coded 1). Third, three separate moderation analyses in only OC users were used to examine whether the masculinity-mental rotations relation varied by pill estrogenic, progestational, or androgenic activity. Age and general cognition were covariates in all analyses, and all predictor variables were centered prior to analyses. Moderations were conducted using the PROCESS macro in SPSS (see Hayes, 2013), with follow-up simple slopes analyses conducted separately for each group (i.e., NC women vs. OC users) or for hormone activity and masculinity scores at the mean plus or minus one standard deviation. Type I error was set at 0.05 for all analyses.

## Results

Prior to testing study hypotheses on the link between masculinity and spatial skills and its potential moderation by hormonal milieu, NC and OC group descriptives, differences, and correlations among study variables were examined. Descriptives are shown on the left of Table 1. Independent *t*-tests revealed no significant differences for general cognition, *t*(368) = 0.81, *p* = 0.421, masculinity, *t*(368) = 0.21, *p* = 0.837, or 3D mental rotations, *t*(368) = −0.08, *p* = 0.934. Correlations for each group are shown on the right of Table 1, revealing consistent expected relations between age and general cognition, and between general cognition and mental rotations. The patterns of relations between masculinity and mental rotations differed across NC women and OC users; these differences are directly tested in moderations below.

**TABLE 1:**
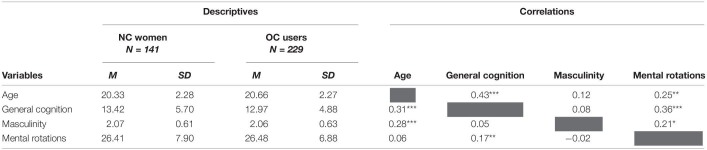
Descriptive statistics and correlations among study variables by group.

Correlations for NC women are above the gray diagonal, and correlations for OC users are below the gray diagonal. NC, Naturally cycling; OC, Oral contraceptive; M, Mean; SD, Standard deviation. *p < 0.05, **p < 0.01, ***p < 0.001.

### Link Between Masculinity and Spatial Skills in All Women

The first inferential analysis examined the link between masculinity and spatial skills in all women. Results of the regression revealed a significant overall model, *F*(3,366) = 9.44, *p* < 0.001, *R*^2^ = 0.07, due to the significant covariate of general cognition, *b* = 0.34, *p* < 0.001; age was not a significant covariate, *b* = 0.14, *p* = 0.431. Importantly, masculinity was not a significant predictor of 3D mental rotations, *b* = 0.59, *p* = 0.331.

### Oral Contraceptive Moderation of the Link Between Masculinity and Spatial Skills

**FIGURE 1:**
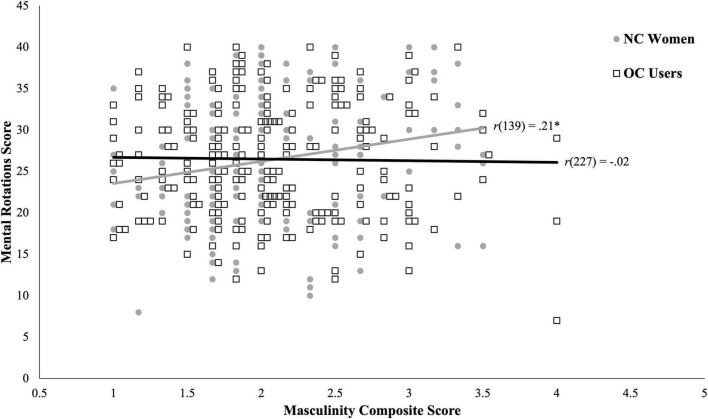
Relation between masculinity and spatial skills (operationalized by three-dimensional mental rotations skill) plotted by hormonal milieu (operationalized by NC vs. OC status). Gray circles and black squares show individual data points of NC women and OC users, respectively, with like-colored lines reflecting the linear trends for each group (i.e., zero-order correlations). NC, naturally cycling; OC, oral contraceptive. *p < 0.05.

The second inferential analysis was a moderation examining whether the link between masculinity and spatial skills differed for NC women and OC users; in other words, it examined whether the lack of an association in the full sample was due in part to women’s hormonal milieu. Results of the regression revealed a significant overall model, *F*(5, 364) = 6.76, *p* < 0.001, *R*^2^ = 0.09. General cognition continued to be a significant covariate, *b* = 0.33, *p* < 0.001, and age was not, *b* = 0.17, *p* = 0.338. Although there was a significant main effect of masculinity, *b* = 2.35, *p* = 0.017 and not NC-OC group, *b* = 0.17, *p* = 0.819, these main effects must be considered in the context of the significant interaction between masculinity and group on 3D mental rotations, *b* = −2.80, *p* = 0.023. Follow-up simple slopes analyses revealed the expected significant positive association for NC women, *b* = 2.35, *p* = 0.017, but not for OC users, *b* = −0.44, *p* = 0.558. The nature of the interaction is also shown in the scatterplot in Figure 1. Individual women are shown by the gray circles (NC women) and black squares (OC users), with the zero-order linear relation for each group indicated by the gray (NC women) and black (OC users) lines.

### Oral Contraceptive Pharmacokinetic Moderation of the Link Between Masculinity and Spatial Skills

**FIGURE 2:**
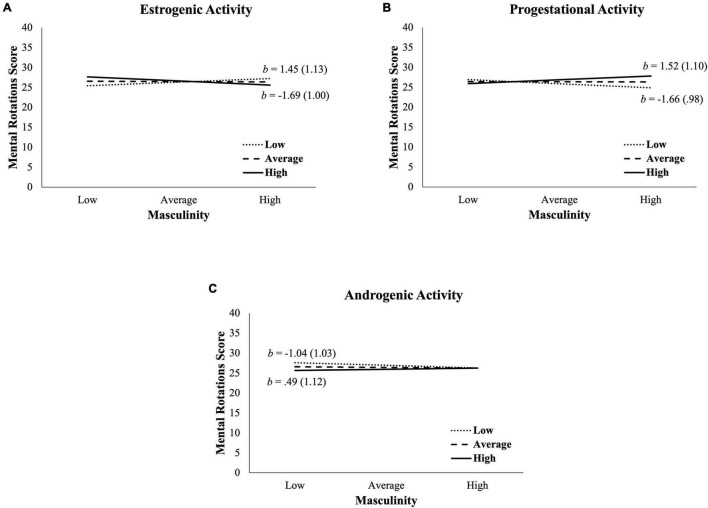
Relation between masculinity and spatial skills (operationalized by three-dimensional mental rotations skill) plotted by oral contraceptive hormone activity (operationalized by a novel 4-point coding scheme). **(A)** The masculinity and spatial skills relation was significantly moderated by OC estrogenic activity. **(B)** The masculinity and spatial skills relation was significantly moderated by OC progestational activity. **(C)** The masculinity and spatial skills relation was not moderated by OC androgenic activity. Dotted, dashed, and solid black lines reflect low, average, and high hormone activity, respectively. Low vs. high hormone activity and masculinity reflect the conditional effect for the model estimated for scores ± one standard deviation from the mean, respectively. Unstandardized coefficients (*b*) reflect the masculinity-mental rotations relation for the low and high hormone activity levels with standard errors in parentheses. See Table 2 for results of statistical interactions.

The third inferential analysis was a moderation among only OC users examining whether the link between masculinity and spatial skills depended upon the pharmacokinetic properties of the pills. Similar to the previous moderation, it examined whether the lack of an association among OC users was due in part to the heterogeneity of OCs, specifically their estrogenic, progestational, and androgenic activity. Complete results are listed in Table 2 and plotted in Figure 2, and significant findings are discussed here.

**TABLE 2:** Results of moderation analyses examining whether sex hormone activity in OCs moderates the link between masculinity and mental rotations skill.

Moderation model	*b*	*F*	df	*R* ^2^
**Estrogenic activity**		2.12^+^	5, 214	0.05
Age	0.04			
General cognition	0.19^+^			
Masculinity	–0.12			
Estrogenic activity	0.14			
Interaction	−1.52*			
**Progestational activity**		2.45*	5, 214	0.05
Age	–0.07			
General cognition	0.20*			
Masculinity	–0.07			
Progestational activity	0.38			
Interaction	1.23*			
**Androgenic activity**		1.61	5, 214	0.04
Age	0.03			
General cognition	0.22*			
Masculinity	–0.27			
Androgenic activity	–0.41			
Interaction	0.65			

N = 220 OC users, and unstandardized b’s are shown in table. ^+^p < 0.10; *p < 0.05.

For estrogenic activity (top third of Table 2), the overall model of the relation between masculinity and mental rotations skill was not significant, but the interaction of masculinity and estrogenic activity was significant. Estrogenic activity at one standard deviation below the mean (dotted line in Figure 2A) was positively related to masculinity and 3D mental rotations, *b* = 1.45, *p* = 0.201, whereas estrogenic activity one standard deviation above the mean (solid line in Figure 2A) was inversely related to masculinity, *b* = −1.69, *p* = 0.094. Thus, the masculinity-mental rotations relation became increasingly positive as levels of OC estrogenic activity *decreased*.

For progestational activity (middle third of Table 2), the overall model was significant. General cognition was a significant, positive covariate, and the interaction of masculinity and progestational activity was significant, with effects in the opposite direction of estrogenic activity. Progestational activity at one standard deviation below the mean (dotted line in Figure 2B) was inversely related to masculinity and 3D mental rotations, *b* = −1.66, *p* = 0.093, whereas progestational activity one standard deviation above the mean (solid line in Figure 2B) was positively related to masculinity, *b* = 1.52, *p* = 0.169. Thus, the masculinity-mental rotations relation became increasingly positive as levels of OC progestational activity *increased*.

For androgenic activity (bottom third of Table 2), the overall model was not significant. The interaction of masculinity and androgenic activity was also not significant, but general cognition was a significant covariate (see Figure 2C).

Sensitivity analyses were conducted to determine whether length of OC use altered these relations; thus, moderations were repeated with length of OC use (in months) as a covariate. The pattern of results, including inferences about the significance of the interactions, did not change (estrogenic activity: *b* = −1.51, *p* = 0.039, progestational activity: *b* = 1.17, *p* = 0.035, androgenic activity: *b* = 0.64, *p* = 0.315).

## Discussion

The goal of this study was to leverage a natural experiment—varying hormonal milieus through OC use—to reveal biopsychosocial links to spatial skills in women. Based on research showing that masculinity and the progestin androgenicity of OCs are independently and positively associated with those skills, this study aimed to—for the first time—examine their combined influence. In a large sample of NC women and OC users, whose pill pharmacokinetic properties were innovatively coded on a four-point scale for estrogenic, progestational, and androgenic activity (see Dickey, 2020), hormonal milieu was found to moderate the relation between self-perceived masculinity and 3D mental rotations skill. Specifically, there was a positive relation for NC women and virtually no relation for OC users. The lack of an association among the heterogenous group of OC users was partly due to the varying pharmacokinetic properties of OCs, as the masculinity-mental rotations relation increased with both decreasing pill estrogenic activity and with increasing pill progestational activity. In contrast, androgenic activity of OCs was not significantly related to the relation between masculinity and mental rotations skill.

### Interpretation of Findings

Across the full sample (i.e., NC women and OC users combined), there was not a significant relation between masculinity and spatial skills. Even though a significant relation was expected according to the sex-role mediation hypothesis (Nash, 1979), the non-significance is not necessarily at odds with the extant literature. Past empirical studies have led to somewhat inconsistent results by statistically controlling for gender and even occasionally finding a stronger relation in men than women (Reilly and Neumann, 2013; Reilly et al., 2016). Importantly, results of moderation analyses qualify that non-significant masculinity-spatial skills relation in revealing that it depends upon ovarian hormonal milieu (marked by NC vs. OC status): Masculinity was significantly and positively related to mental rotations skill for NC women, but not for OC users. The robustness of this interaction effect is buttressed by the fact that NC women and OC users did not differ in mean levels in any study variables, including gender self-concept or mental rotations skill, consistent with past research in heterogenous samples (e.g., Beltz et al., 2015; Nielson and Beltz, 2021). Thus, the sex-role mediation hypothesis may only describe women with some hormonal milieus (e.g., those experiencing the typical fluctuations in endogenous ovarian hormones that characterize the menstrual cycle). As past studies on the sex-role mediation hypothesis did not report the hormonal milieu of study participants (e.g., whether all women were NC), it is possible that varying hormonal milieus contributed to their inconsistent findings. Additionally, varying hormonal milieus may even contribute to the inconsistent findings among men for whom the masculinity-spatial skills relation is not consistently found (e.g., Signorella and Jamison, 1986; Kelly and Beltz, under review), and who experience fluctuations in endogenous sex hormones levels depending upon season of the year, life experiences like fatherhood, and even daily experiences like winning a game (e.g., Zilioli and Watson, 2012; Smith et al., 2013; Grebe et al., 2019).

To directly investigate the extent to which ovarian hormonal milieus may have contributed to the null masculinity-spatial skills relation in OC users, the OCs of all users in the study were coded on a four-point Likert scale for their estrogenic, progestational, and androgenic activity; the codes were based on assays conducted in rodent models and human tissue response, largely reflecting the action of OCs at receptors in the context of other hormones (see Dickey, 2020). Moderation analyses then revealed that the relation between masculinity and 3D mental rotations depended upon both the estrogenic and progestational—but not significantly upon the androgenic—activity in OCs, although effects were clearly small (as seen in Figure 2) and require replication. Specifically, as OC estrogenic activity decreased but as progestational activity increased, there was an increasingly positive association between masculinity and spatial skills. Findings are consistent with the inverse association between OC estrogenic and progestational activity in this sample. Also, findings regarding estrogenic activity are generally consistent with past work among OC users in showing an inverse association between pill estradiol dose and spatial skills (Beltz et al., 2015), and are also consistent with past menstrual cycle studies showing an inverse relation between endogenous estradiol and spatial skills (Hausmann et al., 2000; Courvoisier et al., 2013; Hampson et al., 2014; Griksiene et al., 2018, 2019), as well as with studies that show improvements in spatial skills when estradiol levels are low (Maki et al., 2002; Courvoisier et al., 2013).

Findings regarding progestational activity are novel, as there is hardly any consideration of the effects of the progestins in OCs—beyond their androgenicity—in the extant literature (for an exception and null findings confounded by androgenicity, see Beltz et al., 2015). Furthermore, findings from past research on endogenous progesterone (e.g., from menstrual cycle studies that often have notable methodological limitations, such as small sample size) are mixed (reporting null, positive, and inverse associations; Hausmann et al., 2000; Maki et al., 2002; Griksiene and Ruksenas, 2011; Courvoisier et al., 2013; Hampson et al., 2014; Noreika et al., 2014; Pletzer et al., 2019; Shirazi et al., 2021). There does seem to be a potential indirect role for progesterone in spatial skills through attention, though. Progesterone has been linked to the local vs. global processing of visual stimuli (Pletzer et al., 2014), and similar global-local processing notions have been used to explain gender differences in spatial skills, especially mental rotations (Peña et al., 2008; Pletzer et al., 2014).

Therefore, this research is important not only for the empirical findings it uncovered, but also for introducing and illustrating the utility of a new coding scheme for OCs that disentangles the estrogenic, progestational, and androgenic properties of the pills. Past work has highlighted the importance of considering heterogeneity in ovarian hormonal milieus afforded by different OCs (Beltz and Moser, 2020), but most work has accomplished this by classifying users based on the types of hormones in their pills, such as androgenic or anti-androgenic progestins (e.g., Wharton et al., 2008; Griksiene and Ruksenas, 2011; Gurvich et al., 2020) or according to active ingredients specified by the FDA (e.g., Puts et al., 2010; Beltz et al., 2015, 2019). Some studies have also accomplished this by considering the exogenous dose of one hormone in the pills (e.g., ethinyl estradiol) without simultaneously considering other hormones (e.g., Beltz et al., 2015; Griksiene et al., 2018). The novel four-point coding scheme used in this study may be an improvement upon all of these approaches. It is based on the biological activity of the exogenous hormones in animal tissue, and it does not require the creation of inconsistent group classifications that could result in some participants being excluded from analyses (e.g., if they are using an OC that is not widely represented in the sample). Although this study did not have power to detect higher order interactions, this coding scheme permits the examination of interaction effects between different hormone activities in OCs in future studies with even larger samples (e.g., whether progestational and androgenic activity combine masculinity to influence outcomes).

Because OC androgenic activity did not moderate the masculinity-spatial skills relation, findings from this study may seem inconsistent with past work showing that OCs with highly androgenic progestins facilitate spatial skills, whereas those with anti-androgenic progestins reduce spatial skills (Beltz et al., 2015; Griksiene et al., 2018; Gurvich et al., 2020), and with work showing a positive association between endogenous testosterone and spatial skills (Hausmann et al., 2000; Pletzer et al., 2019). Not all studies find links between progestin androgenicity and spatial skills, though (e.g., Griksiene and Ruksenas, 2011), and no studies have investigated potential neuroendocrine modulation of the relation between masculinity and spatial skills. Moreover, and as noted above, the OC groupings used in past research may conflate androgenic and progestational activity by focusing on groups of OC users determined by progestin androgenicity. It could very well be that past work focused on androgenic properties of pills was actually reflecting (at least to some meaningful degree) progestational activity. For instance, OCs containing the anti-androgenic progestin drospirenone have among the highest progestational activity (Dickey, 2020). Such conflation is less likely in this study because of the small correlation between androgenic and progestational activity.

### Study Considerations

Study findings and presumed methodological advances should be considered in light of other characteristics of the sample and approach. Regarding the sample, it is likely not representative of all OC users, as most participants were White, from a Midwestern university community, and six women who were deemed statistical age outliers were excluded from analyses. Women who identified as Asian were also disproportionately underrepresented among OC users (compared to NC women). It is vital that future research on ovarian hormone links to the brain and behavior include increasingly diverse and longitudinal samples to best inform women’s health across the lifespan. Hopefully, such research will be facilitated by the advances in OC-related research methods proposed in this and other recent work (reviewed in Beltz and Moser, 2020; Hampson, 2020).

Regarding the study approach, a common, validated, and reliable measure of spatial skills, specifically the Vandenberg and Kuse (1978) 3D mental rotations test, was used. This test shows an established gender difference (Voyer, 2011; Halpern, 2013; Beltz et al., 2020), making it ideal for this study’s goal of detecting gendered links (via masculinity) to mental rotations performance. From these findings, however, it is not clear what aspect of 3D mental rotations skill is linked to masculinity. Although general cognitive ability can be largely discounted (as it was a covariate in all analyses), other possibilities are not easily parsed, including actual mental visualization, strategy use including global vs. local processing, or working memory interference caused by time constraints (see Peters, 2005; Pletzer, 2014; Boone and Hegarty, 2017). It is also not clear if the pattern of findings would be the same if a different mental rotations test was used, particularly a test that shows smaller average gender differences than does the Vandenberg and Kuse (1978) measure; this includes 2D tests and the Shepard and Metzler (1971) 3D test. Thus, future work aimed at replicating and potentially decomposing mental rotations skill and its links to masculinity in the context of varying hormonal milieus is needed.

Also, the focus of this study was on the overarching hormonal milieu afforded by having a natural menstrual cycle or using OCs with particular pharmacokinetic formulations, both of which holistically reflect neuroendocrine function. This leads to some unique considerations for NC women and OC users. Regarding NC women, we did not include menstrual cycle phases in analyses, as methods for determining them are error-ridden without many repeated assessments (Hampson, 2020; Gloe et al., under review). Nonetheless, study findings are broadly consistent with menstrual cycle research on spatial skills, suggesting small roles for estradiol and progesterone. Regarding OC users, we similarly did not assess active vs. placebo pill phase, as placebo phases and adherence to them vary greatly across users. Moreover, we did not assess time of pill ingestion; it is linked to spikes in bloodstream hormone concentrations, but the spikes have unclear implications for concentrations across days, and the temporal association between bloodstream concentrations and central nervous system function is largely unknown (Jusko, 2017; Hampson, 2020). Thus, future research using valid and reliable measures of cycle phase and investigating the pharmacokinetic properties of OCs on neurally mediated processes is sorely needed.

Moreover, this study—like all complex biopsychosocial research on OCs—has some unique considerations. For instance, this study was novel in leveraging a coding scheme for estrogenic, androgenic, and progestational activity of OCs, there are undoubtedly individual differences in the biopsychosocial impacts and correlates of these exogenous hormones, as humans are likely more complex than the animal models upon which the coding scheme is based. Thus, there is a pressing need for future work that considers individual women and their daily OC use across time (e.g., intensive longitudinal studies, such as Weigard et al., 2021; Gloe et al., under review).

Finally, the vast majority of the extant research on the sex-role mediation hypothesis (and on gender, more broadly) has operationalized *masculinity* in terms of explicitly defined gendered personality characteristics (e.g., independent vs. patient), which are to some extent culturally determined and time-varying (for a meta-analysis, see Donnelly and Twenge, 2017). In the present study and consistent with established research (e.g., Egan and Perry, 2001; Beltz et al., 2021; Nielson and Beltz, 2021; Kelly and Beltz, under review), masculinity was operationalized by gender self-expression, with its meaning (and the meaning of femininity) being implicitly defined by the participant. Gendered personality qualities and self-expressions are certainly related, but they are distinct constructs with distinct outcomes (Eagly and Wood, 2017; Hyde et al., 2019). Thus, future biopsychosocial research will likely benefit from having multidisciplinary teams who grapple with psychosocial measurement alongside biological indices of hormone activity.

## Conclusion

This biopsychosocial study examined the sex-role mediation hypothesis in the context of women’s ovarian hormonal milieus, revealing that exogenous hormones in OCs moderate the relation between self-perceived masculinity and 3D mental rotations skill: There was a positive relation for NC women and women using OCs with low estrogenic and high progestational activity, but not for women using OCs with intermediate exogenous hormone activity. Moreover, the androgenic activity of OCs was not a significant moderator of the masculinity-spatial skills relation, which was unexpected but plausible, as androgenic and progestational activity were likely confounded in past research. Findings are important not only in delineating the ways in which biological (i.e., ovarian hormone milieu) and psychosocial (i.e., self-perceived masculinity) factors combine to explain variation in spatial skills among women, but also in demonstrating the utility of a new approach for indexing the hormone activity of OCs. In these innovative ways, this study may propel forward research on women’s health, particularly the neurocognitive and behavioral correlates of ovarian hormones.

## Data Availability Statement

The datasets presented in this article are not readily available because data use agreements should be established. Requests to access the datasets should be directed to AB, abeltz@umich.edu.

## Ethics Statement

The studies involving human participants were reviewed and approved by the University of Michigan IRB (Health Sciences and Behavioral Sciences). The patients/participants provided their written informed consent to participate in this study.

## Author Contributions

AB conceptualized and directed the study. AL collected the data. AB analyzed the data with critical input from DK and MN. All authors drafted the manuscript, provided critical revisions, and approved the final version.

## Conflict of Interest

The authors declare that the research was conducted in the absence of any commercial or financial relationships that could be construed as a potential conflict of interest.

## Publisher’s Note

All claims expressed in this article are solely those of the authors and do not necessarily represent those of their affiliated organizations, or those of the publisher, the editors and the reviewers. Any product that may be evaluated in this article, or claim that may be made by its manufacturer, is not guaranteed or endorsed by the publisher.
